# Efficient Removal of Cr(VI) by TiO_2_ Based Micro-Nano Reactor via the Synergy of Adsorption and Photocatalysis

**DOI:** 10.3390/nano12020291

**Published:** 2022-01-17

**Authors:** Yu Song, Xi Lu, Zhibao Liu, Wenfei Liu, Ligang Gai, Xiang Gao, Hongfang Ma

**Affiliations:** 1 School of Environmental Science and Engineering, Qilu University of Technology (Shandong Academy of Sciences), Jinan 250353, China; 1043119575@stu.qlu.edu.cn; 2 Engineering & Technology Center of Electrochemistry, School of Chemistry and Chemical Engineering, Qilu University of Technology (Shandong Academy of Sciences), Jinan 250353, China; Lahshiy@outlook.com (X.L.); 15853177979@163.com (Z.L.); liganggai@126.com (L.G.); 3 Department of Chemistry and Biochemistry, University of California, Los Angeles, CA 90095, USA; wenfei95@gmail.com

**Keywords:** Cr(VI) removal, ZIF-8@TiO_2_, photocatalytic activity, adsorption, methyl orange

## Abstract

The low-toxicity treatment of chromium-containing wastewater represents an important way of addressing key environmental problems. In this study, a core-shell structural ZIF-8@TiO_2_ photocatalyst was synthesized by a simple one-step hydrothermal method. The obtained composite photocatalyst possessed improved photocatalytic activity compared with TiO_2_. The results indicated that the optimized ZIF-8@TiO_2_ composite exhibited the highest removal efficiency with 93.1% of Cr(VI) after 120 min under UV-vis irradiation. The removal curves and XPS results indicated that the adsorbed Cr(VI) on the ZIF-8 during the dark process was preferentially reduced. The superior removal efficiency of ZIF-8@TiO_2_ is attributed to the combination of both high adsorption of ZIF-8, which attracted Cr(VI) on the composite surface, and the high separation efficiency of photo-induced electron-hole pairs. For the mixture of wastewater that contained methyl orange and Cr(VI), 97.1% of MO and 99.7% of Cr(VI) were removed after 5 min and 60 min light irradiation, respectively. The high removal efficiency of multiple pollutants provides promising applications in the field of Cr(VI) contaminated industrial wastewater treatment.

## 1. Introduction

Water pollution has become increasingly severe following the development of industry, attracting widespread attention [1]. Chromium is one of the most dangerous pollutants in water, and it mainly exists in electroplating, petrochemical industry, leather processing, metallurgy, and other industrial wastewaters. As the most common existent form of chromium in wastewater, Cr(VI) has high toxicity and is harmful to the ecological environment and humans. Therefore, an effective method for the treatment of Cr(VI) in wastewater is necessary and has become a hot issue [2,3].

Adsorption and photocatalysis [4,5,6] have been used to remove Cr(VI) from wastewater due to their sustainability, high efficiency, and reasonable cost. Metal-organic frameworks (MOFs) have been widely used as adsorbents to remove pollutants from water due to their high surface area, large pore size, tunability, and suitable nanoscale cavities [7]. For example, silver-triazolate MOFs could adsorb Cr(VI) via anion exchange reaction with a maximal adsorption capacity of 37.0 mg/g at 303 K [8]. The water-stable cationic MOFs could serve as excellent single-crystal containers to capture Cr(VI) [9]. MOFs doped alginate beads also exhibited high Cr(VI) removal efficiency [10]. Although many successes have proven that MOFs are effective adsorbents to remove Cr(VI) from wastewater, the existing form of the adsorbed chromium is still a potential hazard to the environment. 

Photocatalysis applied to reduce Cr(VI) to Cr(III) has gradually attracted the attention of researchers considering the degradation of pollutants from toxic material into low toxicity or nontoxic substances [11,12,13,14,15]. The photocatalyst can convert optical illumination into chemical energy to oxidize and reduce the target substance in the environment. The substance is eventually degraded by free radicals generated by electron and hole pairs that are photo-induced from photocatalysts [16]. TiO_2_, as a commonly used photocatalyst, has been widely used in the pollutant treatment field [17,18,19]. However, TiO_2_ suffers from the limitation of its relatively large bandgap (approximately 3.2 eV for anatase and 3.0 eV for rutile) and low quantum efficiency due to the quick recombination of photo-induced electrons and holes [20,21]. Therefore, enhancing the photoexcitation and separation efficiency of photo-induced electron-hole pairs is important for industrial applications [22,23].

The separation rate of photo-induced electron-hole pairs is a critical factor that directly influences photocatalytic efficiency. The heterostructure is the most commonly used method to improve the separation efficiency of photo-induced electron-hole pairs [24,25,26]. The adsorption performance of the heterostructure also affects photocatalytic efficiency by increasing the effective concentration of pollutants. Thus, the fabrication of a heterostructure with excellent adsorption performance may lead to evident an improvement in the removal efficiency of chromium from wastewater. Among typical MOFs material, ZIF-8 is widely used as pollutant adsorbent, also exhibiting excellent photocatalytic performance. ZIF-8 can attract surrounding pollutants on its surface and inner structure, and thus increase the effective concentration. A heterostructure combined with such MOFs material can improve the charge carrier separation during photocatalysis. The combination of ZIF-8 and TiO_2_ (ZIF-8@TiO_2_) could achieve better removal efficiency of pollutants by adsorption and photocatalysis. The photocatalytic efficiency could be improved due to the pre-enrichment of the pollutants [27].

Herein, a novel heterostructure photocatalyst was synthesized by loading TiO_2_ nanoparticles on a ZIF-8 surface via the hydrolysis method. Many methods were followed to characterize the chemical composition and microstructure of the novel material. The synthesized TiO_2_@ZIF-8 composites were further used to achieve photocatalytic Cr(VI) reduction. In addition, the possible mechanisms of Cr(VI) reduction were proposed.

## 2. Materials and Methods

### 2.1. Reagents and Materials

TiO_2_, 2-Methylimidazole, and methyl orange (MO) were purchased from Shanghai Macklin Biochemical Co., Ltd., Shanghai, China and zinc acetate dihydrate from Xilong Science Co., Ltd., Shantou, China. K_2_Cr_2_O_7_ was purchased from Tianjin Guang Cheng Chemical Reagent Co., Ltd., Tianjin, China. Methanol, anhydrous ethanol, and phosphoric acid were provided by Sinopharm Chemical Reagents Co., Ltd., Shanghai, China and C_13_H_14_N_4_O was provided by Tianjin Kemei Chemical Reagent Co., Ltd., Tianjin, China. Acetone, hydrochloric acid, and sulfuric acid were purchased from Yantai Yuandong Fine Chemical Co., Ltd., Yantai, China.

### 2.2. Synthesis of ZIF-8

ZIF-8 was subjected to hydrothermal synthesis using an AB liquid mixture. First, 0.583 g of zinc acetate was dissolved into 30 mL methanol as solution (A); 0.670 g of 2-methylimidazole was dissolved in 30 mL methanol as solution (B). Second, the solution (B) was slowly added into solution (A) under the condition of stirring. Third, the mixture was introduced into a Polytetrafluoroethylene (PTFE) container and sealed in a stainless-steel container for hydrothermal synthesis at 423 K for 6 h [28]. Fourth, the product was collected by centrifugation, washed several times with water and ethanol, and the sample was freeze-dried to obtain ZIF-8 powder.

### 2.3. Synthesis of ZIF-8@TiO_2_

**Figure 1:**
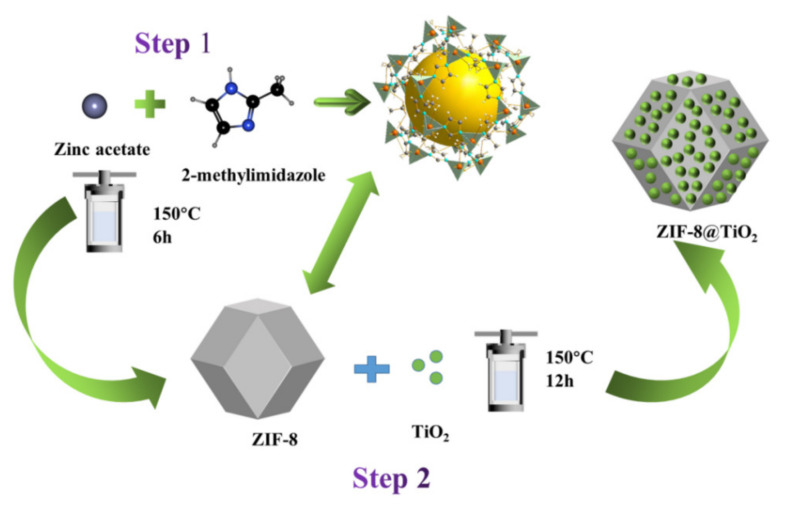
Synthesis process of ZIF-8@TiO_2_ composite, step 1 is the preparation process of ZIF-8, step 2 is the preparation process of ZIF-8@TiO_2_.

The composite of ZIF-8 and TiO_2_ was obtained as follows: 0.1 g of prepared ZIF-8 was stirred in 40 mL ethanol for 20 min. Subsequently, 0.2 g of TiO_2_ was added to the ZIF-8 solution and stirred for 20 min. Then, the mixed solution was transferred to a 100 mL PTFE container and sealed in a stainless-steel reactor. After sealing, the autoclave was heated to 423 K for 12 h [29]. The subsequent treatment of the material was the same as that of ZIF-8. The synthetic route of ZIF-8@TiO_2_ is shown graphically in Figure 1. The composite of ZIF-8 and TiO_2_ is referred to as Z*x*T*y*. Letters “Z” and “T” represent ZIF-8 and TiO_2_, respectively. The “*x*” (200, 150, 100, 75 and 60) and “*y*” (100, 150, 200, 225 and 240) are the mass percentages of ZIF-8 and TiO_2_, respectively.

### 2.4. Cr(VI) Removal Measurement

Cr(VI) wastewater was prepared by dissolving potassium dichromate in deionized water, and the photocatalytic activity was evaluated under the UV-vis irradiation of a 300 W xenon-lamp (200–2500 nm, 1800 mW/cm^2^, Beijing Aulight Cp.,Ltd,. Bejing, China). Then, 20 mg of ZIF-8@TiO_2_ composite was added to a quartz reactor with 60 mL Cr(VI) (20.0 mg/L) solution at room temperature for Cr(VI) removal measurement [30]. Before irradiation, the solution was stirred in the dark for 60 min to achieve adsorption equilibrium and then exposed to ultraviolet light. Cr(VI) concentration was measured by ultraviolet spectroscopy at 540 nm. The following equation determines the removal efficiency:

*Removal efficiency % = (C*_0_*− C*_t_*)/C*_0_*×* 100% (1)

*Proportion of pollutants = C*_t_*/C*_0_ (2)

where *C*_t_ is the Cr(VI) concentration at the time of measurement, and *C*_0_ the initial Cr(VI) concentration.

### 2.5. Photoelectrochemical Measurement

Briefly, 10 mg ZIF-8, TiO_2_, or ZIF-8@TiO_2_ were added into 10 mL ethanol and ultrasonicated for 60 min to ensure that the particles were evenly dispersed in the solution. Hence, 10 mL of the prepared solution were dropped onto the fluorine doped tin oxide (FTO) glass (Jinan Kester Experimental Equipment Co., Ltd., Jinan, China) substrate (exposed area: 1.0 cm^2^) and dried in a vacuum of 333 K for 1 h. This step was repeated to ensure that the FTO glazing catalyst is uniformly covered. The photoelectrochemical measurements were performed using an electrochemical analyzer (CHI-660D) with a three-electrode system. The electrolyte is an aqueous solution of 0.1 M sodium sulfate (pH = 7.0). ZIF-8, TiO_2_, and TiO_2_@ZIF-8 photoelectrodes were used as the working electrodes, Pt plate as the counter electrode, and Ag/AgCl electrode as the reference electrode. The working electrode is irradiated with a 300 W xenon-lamp [31] electrochemical analyzer (CHI-660D). All experiments were conducted at Qilu University of Technology.

### 2.6. Characterization

The crystal textures of the prepared materials were obtained via X-ray diffractometry (XRD, D8-ADVANCE, Bruker AXS, Karlsruhe, Germany) at a scanning angle range of 5–80°. Nitrogen adsorption-desorption isotherm measurements were performed with a high-speed automated surface area and pore size analyzer (Autosorb-iQ-MP, Quantachrome Instruments, Boynton Beach, FL, USA) at 77 K. The surface morphologies of ZIF-8 and Z*x*T*y* were characterized via SEM (JSM-7610F, Shandong Guangdi Testing Technology Co., Ltd., JEOL, Tokyo, Japan). FTIR (IRAffinity-1S WL, Shimadzu Corporation, Tokyo, Japan) in the wavenumber from 400 cm^−1^ to 4000 cm^−1^ was used to show the main characteristics of the functional groups on the Z*x*T*y* surface. The chemical states of the catalyst surface were identified via XPS (ESCALAB Xi+, ThermoFisher, Brno, Czech Republic). 

## 3. Results

### 3.1. Microstructure, Composition, and Performance Characterization of ZxTy 

**Figure 2:**
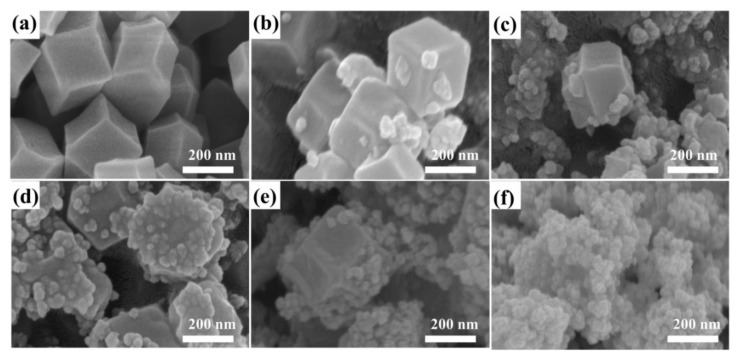
SEM images of (**a**) ZIF-8 and (**b**–**f**) ZIF-8@TiO_2_ composites: (**b**) Z200T100, (**c**) Z150T150, (**d**) Z100T200, (**e**) Z75T225, and (**f**) Z60T240.

The morphology of the synthesized ZIF-8 and Z*x*T*y* samples with different recombination ratios was observed by SEM imaging. As shown in Figure 2a, the ZIF-8 sample exhibited a regular dodecahedron structure with a smooth surface. The side length of the dodecahedron was up to 200 nm. The morphology of Z*x*T*y* composites was evidently varied due to different ratios between ZIF-8 and TiO_2_. When the ratios of ZIF-8 and TiO_2_ were 1:0.5 (Z200T100) and 1:1 (Z150T150), rare TiO_2_ particles appeared on the surface of ZIF-8 (Figure 2b,c). As the ratio increased to 1:2 (Z100T200), as shown in Figure 2d, TiO_2_ particles were successfully coated on the surface of ZIF-8. As the ratio further increased to 1:3 (Z75T225) and 1:4 (Z60T240), TiO_2_ particles presented agglomeration due to the surface effect of nanoparticles (Figure 2e,f). The synthesized Z100T200 composites still maintained the general outline of the dodecahedron with relatively blunt edges and rough surface, which might be beneficial to the subsequent photocatalytic process.

**Figure 3:**
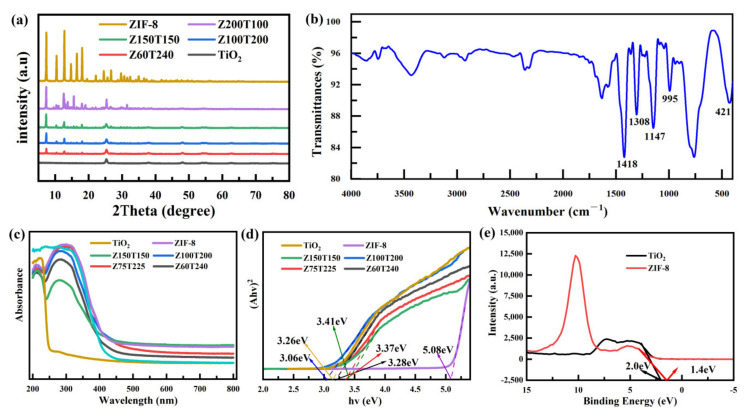
(**a**) XRD patterns, (**b**) FTIR spectrum, (**c**) UV-visible absorption spectra, (**d**) Optical band gap energy plots and (**e**) Valence band XPS spectra of ZIF-8, TiO_2_ or ZIF-8@TiO_2_ (Z*x*T*y*).

XRD analysis was used to investigate the crystal structure of the synthesized ZIF-8, TiO_2_, and Z*x*T*y*. For the synthesized Z*x*T*y* composites, the spectra in Figure 3a exhibited an evident biphasic structure. The peaks located at 7.5°, 10.6°, 12.9°, 16.6°, and 18.2° corresponded to (220), (311), (400), (511), and (440) crystal planes of ZIF-8, respectively [32]. The other peaks at 25.4°, 38.1°, 48.0°, 53.9°, and 55.1° are organized from the (101), (112), (200), (105), and (211) crystal planes of TiO_2_ [33]. According to Figure 3a, no alternative characteristic peaks occur, and the position of the diffraction peaks of ZIF-8 and TiO_2_ barely change. These results suggested that the crystal structures of ZIF-8 and TiO_2_ were unaffected by each other [34]. Meanwhile, the XRD spectra of composites with different reaction ratios between ZIF-8 and TiO_2_ were almost the same, proving that the amount of TiO_2_ did not affect the crystal structures of the composite.

The FTIR spectrum of Z100T200 is shown in Figure 3b. The vibration bands at 1418 cm^−1^ and 995 cm^−1^ originated from the stretching vibration of C-N bond. The other evident peaks located at 1147 cm^−1^ and 1308 cm^−1^ correspond to the bending vibration of imidazole. Another characteristic peak of ZIF-8 at 421cm^−1^ was associated with the stretching vibration of the Zn-N bond. These results further indicated that TiO_2_ was successfully coated on the surface of ZIF-8 [35]. 

Figure 3c shows the UV-vis diffuse reflectance spectra of TiO_2_, ZIF-8, and Z*x*T*y*. As displayed in Figure 3c, the absorption edge of ZIF-8 and TiO_2_ was approximately 250 nm and 410 nm, respectively. For the synthesized Z*x*T*y* composites, the absorption edges were between 420 and 450 nm, which is wider than that of TiO_2_. Among the various composites, the Z100T200 presented the widest absorption edge, which is 450 nm. According to the previous studies [26], the band gap energy (*E_g_*) can be calculated according to the following equation:

(3) $${\left(\alpha h\nu \right)}^{\frac{1}{2}}=A\left(h\nu -E_{g}\right)$$

where *α* is the absorption coefficient, *h* is Planck constant, *ν* is light frequency, *A* is proportionality constant, and *E_g_* is band gap [36,37].

As illustrated in Figure 3d, the band gap widths of ZIF-8 and TiO_2_ were 5.08 eV and 3.26 eV, respectively. When the ratio between ZIF-8 and TiO_2_ was 1:2, the value of the band gap reduced to 3.06 eV. The reduced band gap energy is beneficial to improving photocatalytic efficiency. Thus, the results indicated that the photocatalytic efficiency of Z100T200 was higher than the bare TiO_2_, which might exhibit the highest efficiency.

The valence band (VB) XPS spectra of the as-prepared TiO_2_ and ZIF-8 are demonstrated in Figure 3e. The measured VB edge positions of TiO_2_ and ZIF-8 were at 2.0 eV and 1.4 eV, respectively. Thus, the conduction band (CB) edge positions of TiO_2_ and ZIF-8 were −1.26 eV and −3.68 eV, respectively [38].

**Figure 4:**
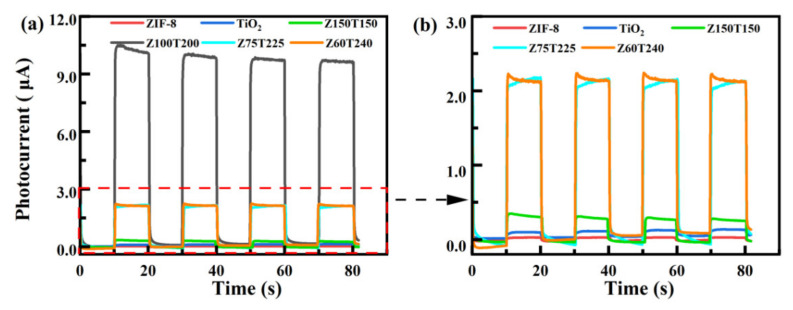
(**a**) Transient photo-current responses of ZIF-8, TiO_2_, and ZIF-8@TiO_2_ (Z*x*T*y*); (**b**) Partial enlarged drawing of (**a**).

Photocurrent tests were performed under intermittent light illumination to investigate the separation efficiency of photo-induced electrons and holes. The results are illustrated in Figure 4. As shown in Figure 4b, the photo-current intensities of bare ZIF-8 and TiO_2_ were 0.1 μA and 0.3 μA, respectively. The ratio between the ZIF-8 and TiO_2_ in the composites could affect the photo-current intensities. When the ratio was 1:1 (Z150T150), the current intensity was higher than the bare TiO_2_. When the ratio increased to 1:3 (Z75T225) and 1:4 (Z60T240), the current intensities were improved. According to Figure 4a, the photo-current intensity of Z100T200 was 10 μA, suggesting that the composite ratio of the ZIF-8 and TiO_2_ is not a single factor affecting the performance, which is also related to the morphology and structure of the material. According to Figure 2, it could be inferred that when the ratio was 1:2 (Z100T200), the TiO_2_ showed a more uniform load. The synergistic combination between ZIF-8 and TiO_2_ could weaken the recombination between electrons and holes [39,40]. Thus, the lifetime of photo-induced carriers could be increased. This result was also relatively consistent with the relationship of band gap width of Z*x*T*y* in Figure 3d, further confirming that Z100T200 has superior catalytic performance.

### 3.2. Removal of Cr (VI)

**Figure 5:**
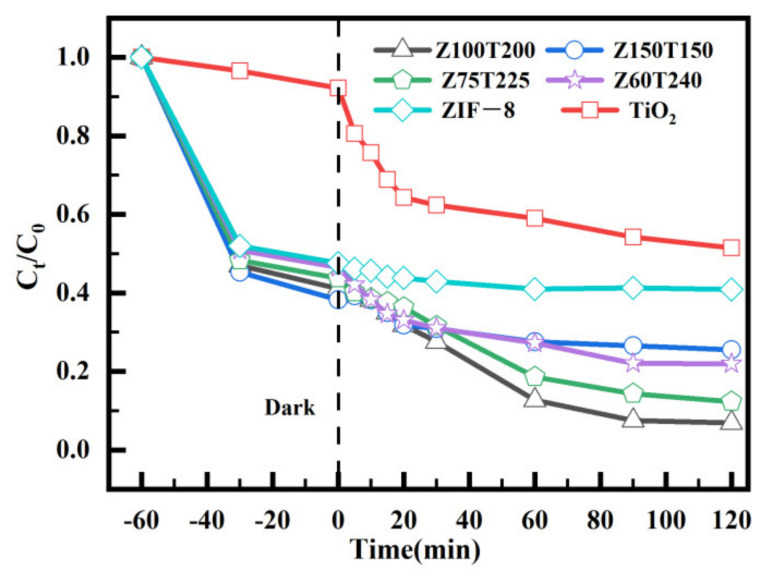
Removal efficiencies for Cr(VI) of, TiO_2_, and ZIF-8 and ZIF-8@TiO_2_(Z*x*T*y*).

**Figure 6:**
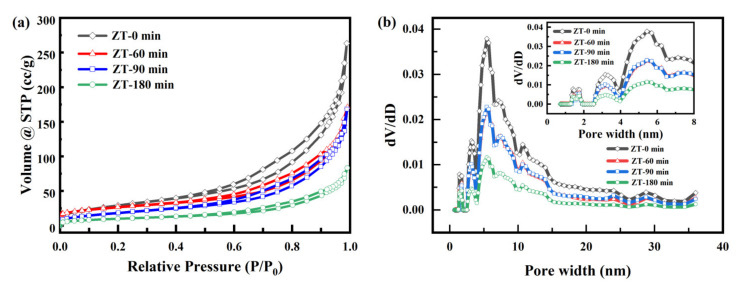
N_2_ adsorption/desorption isotherms (**a**) and pore size distribution (**b**) of Z100T200 at different catalytic time.

As shown in Figure 5, the photocatalytic properties of all materials were tested. The removal efficiency of pure TiO_2_ reached 30% after 30 min of illumination. Due to the porous structure, ZIF-8 showed a good adsorption effect in the dark reaction stage, and the removal rate could reach 50%. When the lamp was turned on, the degradation efficiency of ZIF-8 tends to be flat and reach dynamic equilibrium. These results indicated that pure ZIF-8 has no obvious light response characteristics, which is also consistent with the transient photocurrent response results in Figure 4. ZIF-8@TiO_2_ composite showed better treatment effects than ZIF-8 and TiO_2_. In the dark reaction stage, all composites showed a similar removal trend, and the removal efficiencies were all higher than that of ZIF-8 (50%). This indicates that the composite retains the active specific surface area of ZIF-8. During the photocatalytic process, Z100T200 showed the best removal effect, with the removal efficiency of up to 93%, which is consistent with the band-gap width test in Figure 3 and the transient photocurrent response structure in Figure 4. In Figure 5, the slope of the curve represents the reaction rate. In the first 10 min after the illumination, the reaction rate of the composite material is relatively low, about 0.8 mg/min, and then the reaction rate begins to rise. Cr(VI) was enriched during the dark reaction due to the porous structure of the material. Once the lamp was turned on, the material gives priority to the catalytic degradation of the absorbed Cr(VI), resulting in little change of Cr(VI) concentration in water. When this process is over, the material continues to absorb and catalyze Cr(VI), and the reaction rate gradually accelerates. With the increase of illumination time, more and more Cr(VI) is enriched and converted into Cr(III), which gradually fills the porous structure of the composite, leading to a gradual decrease in the reaction rate. This can also be seen from the specific surface area and pore size distribution of materials at different reaction stages in Figure 6. The Cr(VI) removal capacity by various photocatalytic materials is summarized in Table 1, which indicates that the Z100T200 shows better catalytic performance.

For Z100T200, the band gap reduced compared with TiO_2_, leading to higher photocatalytic property [42]. Meanwhile, the photo-electrons at CB of TiO_2_ could transfer to HOMO of ZIF-8 to recombine with the holes according to the VB information. Therefore, the photo-electrons of the Z100T200 composites mainly existed at the LUMO of ZIF-8. ZIF-8 could adsorb abundant Cr(VI) during the dark process. Thus, for Z100T200, the concentration of Cr(VI) on ZIF-8@TiO_2_ was higher than that of the solution. Z100T200 could act as one “micro-nano reactor” to accelerate the reduction reaction between photo-electrons and Cr(VI) due to the pre-enrichment of the Cr(VI). Thus, as illustrated in Figure 5, during the first 5 min of the irradiation, the removal rate of Cr(VI) in the solution was slower than that in the later process. The reason for this may be that the photo-electrons reduced the adsorbed Cr(VI) on ZIF-8 as a priority.

ZIF-8@TiO_2_ + *hν* → ZIF-8@TiO_2_ + *h*^+^ + e^−^ (4)

Cr_2_O_7_^2−^ + 14H^+^ + 6e^−^ → 2Cr^3+^ + 7H_2_O (5)

The changes of specific surface area, pore volume, and pore size of Z100T200 during the 180-min process could also reflect the removal process, and the results are exhibited in Figure 6, Table 2, Figure S1 and Table S1. According to Figure 6a, the outlines of the curves at different stages show almost no modification, revealing that the microstructure of the material barely changed. After the 60-min dark process, the specific surface area decreased evidently, implying that the material has a certain adsorption capacity for Cr(VI). During the 120-min illumination process, the specific surface area and pore volume of Z100T200 exhibited one interesting phenomenon. During the first 30 min of illumination, the specific surface area and pore volume of Z100T200 basically did not change, indicating that the material was still in the process of adsorption and catalysis. After 120 min of illumination, the specific surface area of the material decreased to 36.7 m^2^/g. As can also be seen in the pore size distribution map corresponding to Figure 6b, the overall pore ratio of the material is decreased, and this difference can be counted as the activity ratio surface area of the material, approximately about 70.3 m^2^/g. The molecular volume of Cr(VI) was larger than that of Cr(III). During the 120-min illumination process, mainly Cr(VI) was reduced to Cr(III). More Cr(III) was adsorbed into the Z100T200, resulting in decreased specific surface area and pore volume. The specific degradation process can be referred to Equations (4) and (5). Under light illumination, the Z*x*T*y* could produce electrons and holes. The photoelectron could act as reducing agent and Cr(VI) is one strong oxidant. Cr(VI) could be reduced to Cr(III) through the redox reaction between Cr(VI) and photoelectron. Meanwhile, the reclaimed catalyst was light green, which was consistent with the color of Cr(III), proving that the Cr(VI) was mainly reduced to Cr(III) and Z100T200 could adsorb Cr(III).

**Figure 7:**
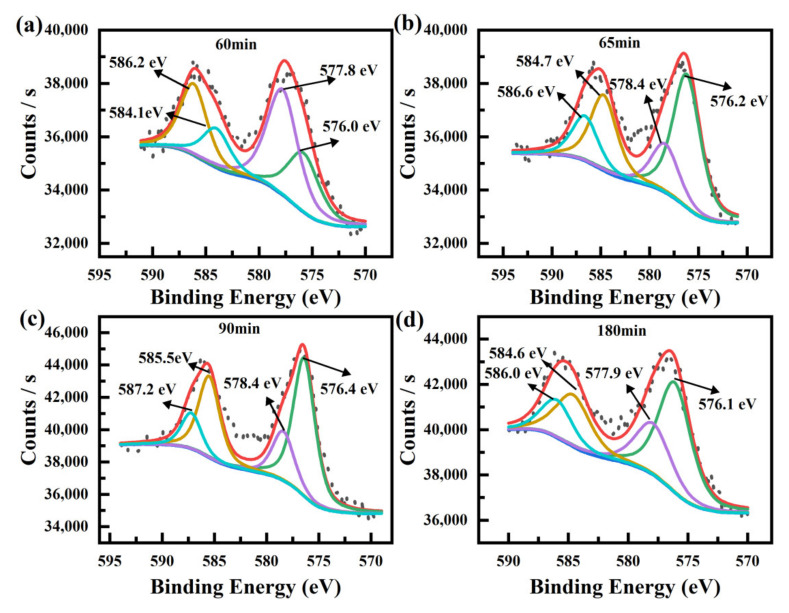
Core-level XPS of Cr for Z100T200 at different stages during the removal process. (**a**) 60 min. (**b**) 65 min. (**c**) 90 min. (**d**) 180 min.

The XPS technique is used to analyze the chemical composite during the removal process to further confirm the reaction process and the results are shown in Figure 7 and Table 3. As illustrated in Figure 7a, after the 60-min dark process, the Cr2p region could be fitted into two pairs of peaks. The peaks located at low binding energy (576.0 and 584.1 eV) correspond to Cr(III), and the other pair peaks at high binding energy (577.8 and 586.2 eV) originated from Cr(VI). The appearance of peaks corresponding to Cr(III) indicated that the composite exhibited a weak catalytic effect under dark conditions. After 5-min irradiation, the valence state of adsorbed chromium was evidently changed. According to the fitting parameters of the XPS results listed in Table 2, the atomic ratio between Cr(VI) and Cr(III) was transformed from 1.73 to 0.37, indicating that almost 30% of the contents of Cr(VI) was reduced to Cr(III) during the five minutes. Meanwhile, as demonstrated in Figure 5a, during the first 5 min of the irradiation process, the concentration of Cr(VI) in the solution remained constant. These results demonstrate that during the initial stage of the irradiation [43,44], the reduced reaction due to the photo-electrons occurred at the adsorbed chromium priority. The reasons for this result are as follows: (1) The concentration of the chromium at the surface or pores of ZIF-8 was higher than the solution owing to the adsorption process. Thus, the ZIF-8 could act as a “micro-nano reactor” to accelerate the reaction rate. (2) The quantity and reactivity of the photo-electrons on ZIF-8 were improved due to the fabricated heterostructure between ZIF-8 and TiO_2_. According to the XPS results of the composite after 90-min and 180-min irradiation shown in Figure 7c,d and Table 3, the chemical composition barely changed, indicating that the reduced reaction mainly existed at the material–solution interface. Then, the adsorption process and reduced reaction occurred at the same time. This result was also demonstrated by the photocatalytic degradation curves shown in Figure 5. After the initial 5-min irradiation, the concentration of Cr(VI) in the solution decreased, following the prolonged irradiation time [45,46]. More information concerning the changes of chemical compositions during the whole process, such as XRD and FTIR, is shown in Figures S2 and S3.

**Figure 8:**
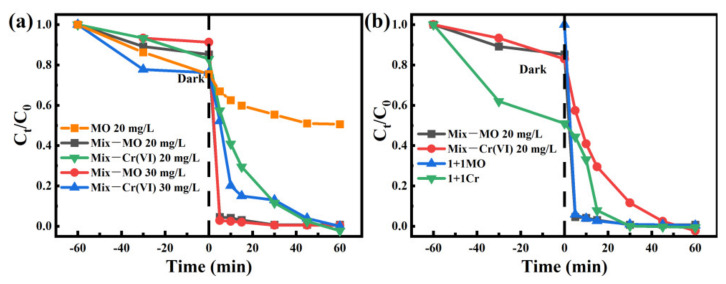
(**a**) Removal efficiencies of MO and Cr(VI) in mixture solution contained MO and Cr(VI); (**b**) The effect of MO addition type on Cr(VI) degradation, the sign of “Mix” indicates that MO and Cr(VI) was added into the solution at the same time, the sign of “1 + 1” indicates that MO was added into the Cr(VI) solution after a 60-min dark process.

Under the irradiation conditions, photo-electrons and holes were produced at the same time. The reduction reaction occurred between the photo-electrons and Cr(VI). The holes were consumed by other organic pollutants, the separation efficiency between photo-electrons and holes and the reduction efficiency of Cr(VI) might be improved. MO was added to the solution to confirm this concept, and the photocatalytic degradation curves are presented in Figure 8. As shown in Figure 8a, when 20.0 mg/L MO was added to the Cr(VI) solution, the adsorption efficiency of Cr(VI) (dark process) was evidently decreased. However, when MO was catalyzed alone, Z100T200 did not show a good catalytic effect. After 60 min of illumination, the removal effect was about 50%. The reaction rate was 0.6 mg/min after ten minutes of illumination. This finding may be due to the competitive adsorption between Cr(VI) and MO. The interesting result was that the photocatalytic efficiency and rate of Cr(VI) were evidently improved due to the addition of MO [47,48]. During the 60-min photocatalytic degradation process, the concentration of Cr(VI) in the solution was 0.3%. The concentration of MO decreased to 2.9% after the 5-min irradiation, with the rate of 3.884 mg/min. When the concentration of MO was increased to 30.0 mg/L, the concentration of Cr(VI) in the solution decreased to 85.0% during the 15-min irradiation process with the rate of 1.13 mg/min, which was faster than that of the concentration of MO 20.0 mg/L (70.5%). More information concerning the influence of the amount of MO on the removal of Cr(VI) is shown in Figure S4. These results indicated that the consumption of holes could improve the degradation efficiency of Cr(VI) [49,50]. The MO was added into the Cr(VI) solution after the 60-min adsorption process to further confirm the role of MO as hole consumer. As indicated in Figure 8b, under this condition, the adsorption efficiency of Cr(VI) was almost the same as that shown in Figure 5 and higher than that of the MO and Cr(VI) mixture solution. Under the 15-min irradiation, the removal efficiency of Cr(VI) reached 92.2%, which was much higher than that of bare Cr(VI) solution (65.0%). Under only 30-min irradiation, the Cr(VI) was degraded completely, while the removal efficiency of bare Cr(VI) was 72.5%. The degradation rate of MO barely changed [51]. These results indicated that the ZIF-8 could act as a nanoreactor to improve photovoltaic efficiency.

### 3.3. Possible Mechanism of Photocatalytic Activity

**Figure 9:**
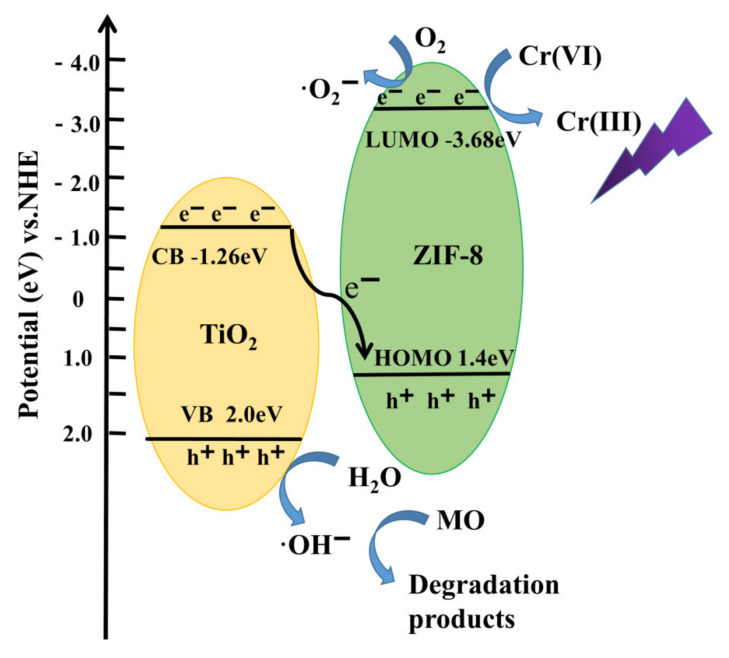
The photo-catalysis mechanism of Cr(VI) and MO and energy-band gap of Z100T200.

In accordance with this discussion, the possible mechanism for the removal of Cr(VI) from the solution is summarized and displayed in Figure 9. ZIF-8 and TiO_2_ could produce photo-electrons and holes under ultraviolet light. The photo-electrons on the CB of TiO_2_ tend to transit to the HOMO of ZIF-8 and further combine with holes [52]. Thus, compared with the bare ZIF-8 and TiO_2_, the separation efficiency of the photo-induced electron-hole pairs for the synthesized heterostructure was improved. The photo-electrons could remain on the LUMO of ZIF-8, and holes could remain on the VB of TiO_2_. Cr(VI) could be easily reduced by the photo-electrons on the LUMO of ZIF-8. Therefore, according to the photocatalytic degradation curves illustrated in Figure 5, the removal efficiency of Cr(VI) was much higher than that of bare ZIF-8 and TiO_2_. Although the separation efficiency of the photo-electrons and holes was improved due to the heterostructure between ZIF-8 and TiO_2_, the photo-electrons and holes can combine with each other, thereby affecting the efficiency of the reduction reaction. The holes on VB of TiO_2_ exhibited excellent oxidization, which could be consumed by organic pollutants. When MO was added into the Cr(VI) solution, the oxidation reaction between the holes and MO could occur on TiO_2_ [53]. The consumption of holes could improve the separation efficiency between photo-electrons and holes, thereby promoting the removal of Cr(VI). This condition may be the reason why the addition of MO to the solution could improve the removal efficiency of Cr(VI). Thus, the pre-adsorption of Cr(VI) on ZIF-8 could improve the photocatalytic efficiency [54,55,56,57]. This speculation could explain why the concentration of Cr(VI) barely changed during the first 5 min of the irradiation process.

## 4. Conclusions

A series of Z*x*T*y* composites were prepared by the hydrothermal method, and Z100T200 showed the best performance. Under UV-vis irradiation, the removal efficiency of Cr(VI) was as high as 93.1%. Photocatalytic degradation experiments and XPS results indicated that the reduction reaction initially occurred at the adsorbed Cr(VI) on the surface of ZIF-8, and the reduction rate during the first 5-min was higher than the subsequent process. These results indicated that ZIF-8 could improve the photocatalytic activity of TiO_2_ and act as a “micro-nano reactor” to increase the rate of photocatalytic Cr(VI) reduction. For the wastewater containing MO and Cr(VI), the removal efficiency of MO was as high as 97.1% after 5 min of illumination, and 99.7% of Cr(VI) was removed after 60 min of illumination. This study introduced an approach to design more ingenious composites which could achieve the goal of multi-pollutant treatment by simple synthetic methods.

## Figures and Tables

**Table 1:** Treatment of Cr(VI) with different photocatalytic materials.

Photo-Catalyst	Cr(VI) Concentration	Lamp Source	Catalyst Dosage	Light Application Time	Removal Rate	Reference
Cu_2_O-Au-TiO_2_	10 mg/L	UV-vis light	1 g/L	60 min	92%	[2]
Cu_2_O-Au-TiO_2_	10 mg/L	visible light	1 g/L	180 min	43%	[2]
MoS_2_/TiO_2_	10 mg/L	visible light	1 g/L	480 min	99.57%	[4]
Mn-TiO_2_ (rGO)	20 mg/L	sunlight	1 g/L	60 min	99.02%	[11]
CDs/MT	10 mg/L	LED-light	1 g/L	30 min	100%	[17]
TiO_2_@ZIF-8	20 mg/L	UV-vis light	0.5 g/L	60 min	99%	[33]
MIL-53(Fe)/Bi_12_O_17_Cl_2_	10 mg/L	white light	0.5 g/L	90 min	99.2%	[41]
Z100T200	20 mg/L	UV–vis light	0.34 g/L	60 min	97.1%	In this study

**Table 2:** S_BET_, pore volume and average pore diameter of Z100T200 at different catalytic time.

Sample	S_BET_ (m^2^/g)	Pore Volume (cm^3^/g)	Average Pore Diameter (nm)
ZT-0 min	107.0	0.407	15.223
ZT-60 min	68.0	0.252	1.482
ZT-90 min	67.9	0.260	1.533
ZT-180 min	36.7	0.128	1.402

**Table 3:** Parameters obtained from XPS shown in Figure 7.

Cr(III)			Cr(VI)		
Samples	Cr 2p_1/2_ (eV)	Cr 2p_3/2_ (eV)	Cr 2p_1/2_ (eV)	Cr 2p_3/2_ (eV)	Area Ratio of Cr(VI)/Cr(III)
K_2_Cr_2_O_7_			588.6	579.4	
Cr_2_O_3_	586.2	576.5			
Z100T200-60 min	584.1	576.0	586.2	577.8	1.73
Z100T200-65 min	584.7	576.2	586.6	578.4	0.37
Z100T200-90 min	585.5	576.4	587.2	578.4	0.36
Z100T200-180 min	584.6	576.1	586.0	577.9	0.50

## Data Availability

The data presented in this study are available on request from the corresponding author.

## Supplementary Materials

The following are available online at https://www.mdpi.com/article/10.3390/nano12020291/s1, Figure S1: N2 adsorption/desorption isotherms of ZIF-8 and Z100T200, Figure S2: (a) XRD-Cr(VI) and (b) XRD-Cr(III) atlases of Z100T200 at different catalytic, Figure S3: FITR-Cr(VI) atlases of Z100T200 at different catalytic stages, Figure S4: Effect of different concentrations of MO on the removal efficiency of Cr (VI), Table S1: SBET, pore volume and average pore diameter of ZIF-8 and Z100T200.
