# Supplementary material for: Efficient Removal of Cr(VI) by TiO2 Based Micro-Nano Reactor via the Synergy of Adsorption and Photocatalysis
Source: Nanomaterials (Basel). 2022 Jan 17;12(2):291. doi: 10.3390/nano12020291 (PMC8778119; doi:10.3390/nano12020291)
Supplement: Supplementary file 1 [file nanomaterials-12-00291-s001.zip › nanomaterials-1550603-supplementary.pdf]

Supplementary Materials

Efficient Removal of Cr(VI) by TiO_2 Based Micro-Nano Reactor via the Synergy of Adsorption and Photocatalysis

Yu Song [1], Xi Lu [2], Zhibao Liu [2], Wenfei Liu [3], Ligang Gai [2], Xiang Gao [2,*] and Hongfang Ma [1,2,*]

[1] School of Environmental Science and Engineering, Qilu University of Technology (Shandong Academy of Sciences), Jinan 250353, China; 1043119575@stu.qlu.edu.cn

[2] Engineering & Technology Center of Electrochemistry, School of Chemistry and Chemical Engineering, Qilu University of Technology (Shandong Academy of Sciences), Jinan 250353, China; Lahshiy@outlook.com (X.L.); 15853177979@163.com (Z.L.); liganggai@126.com (L.G.);

[3] Department of Chemistry and Biochemistry, University of California, Los Angeles, CA 90095, USA; wenfei95@gmail.com

* Correspondence: gaoxiang01@qlu.edu.cn (X.G.); mhf@qlu.edu.cn (H.M.)

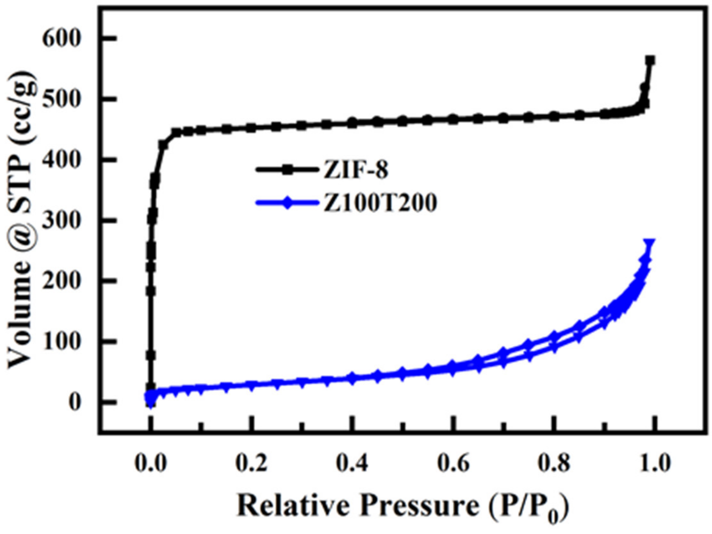

Figure S1. N_2 adsorption/desorption isotherms of ZIF-8 and Z100T200

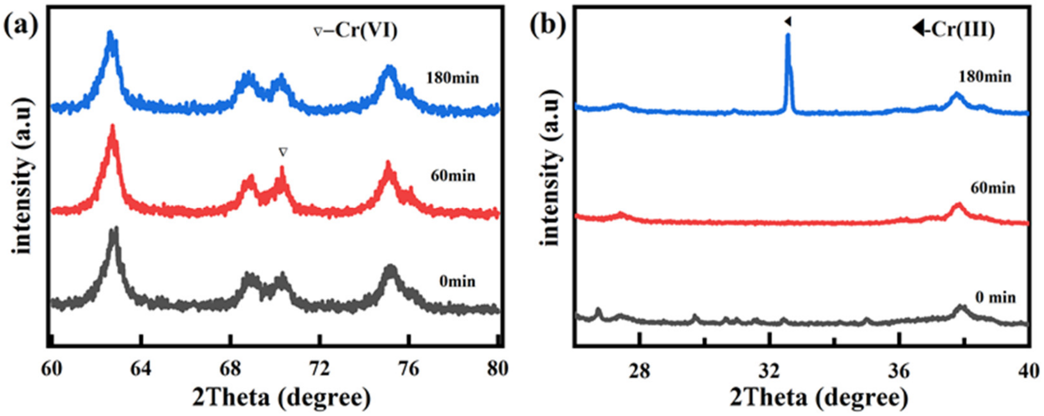

Figure S2. (**a**) XRD-Cr(VI) and (**b**) XRD-Cr(III) atlases of Z100T200 at different catalytic stages.

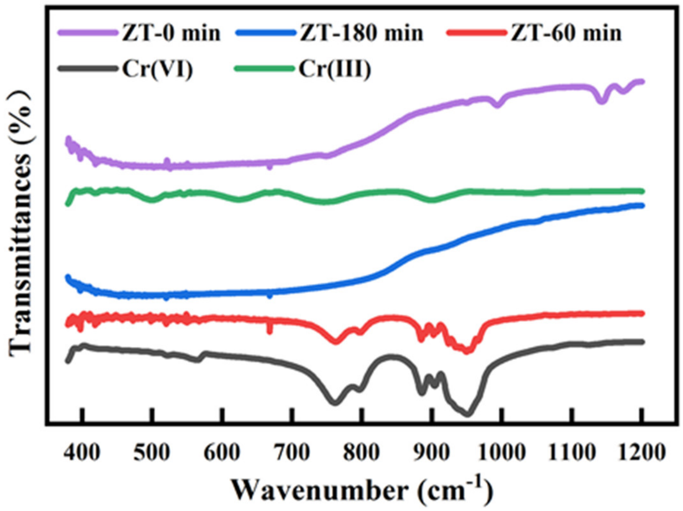

Figure S3 FITR-Cr(VI) atlases of Z100T200 at different catalytic stages.

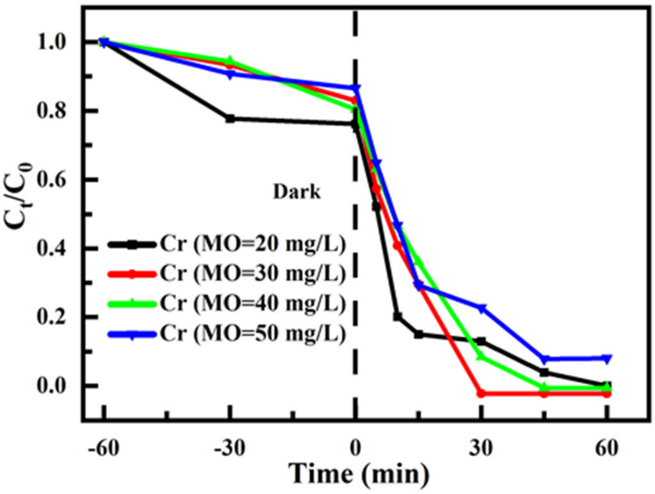

Figure S4 Effect of different concentrations of MO on the removal efficiency of Cr (VI).

Table S1. S_{BET}, pore volume and average pore diameter of ZIF-8 and Z100T200.

Sample	S_{BET} (m^2/g)	Pore volume (cm^3/g)	Average pore diameter (nm)
ZIF-8	1899.6	0.872	1.837
Z100T200	107.0	0.407	15.223
